# Effect of nanostructural irregularities on structural color in the tail feathers of the Oriental magpie *Pica serica*

**DOI:** 10.1371/journal.pone.0282053

**Published:** 2023-03-22

**Authors:** Sangkyu Park, Jihoon Choi, Bohyun Kim, Heeso Noh, Sang-im Lee

**Affiliations:** 1 Department of Physics, Kookmin University, Seoul, South Korea; 2 Smart Natural Space Research Centre, Kongju National University, Cheonan, South Korea; 3 Department of New Biology, DGIST, Daegu, South Korea; University of Akron, UNITED STATES

## Abstract

The tail feathers of magpies are iridescent, with hues ranging from navy to violet and green. It has been previously shown that the hexagonal arrangement of melanosomes in the distal barbules is responsible for these colors, but previous simulation models have relied on average values for the parameters associated with this arrangement (e.g., periodicity), and it remains to be studied whether the actual (rather than averaged) structural arrangement and its inherent irregularities reliably predict structural color. Previous studies using unmodified images for the analysis have not focused on the effect of such irregularities on the color production. In this study, we conducted finite-difference time-domain (FDTD) simulations using actual transmission electron microscopy (TEM) images obtained from the distal barbules of a magpie tail feather, compared the reflectance spectra predicted using the FDTD simulation with those measured with a spectrometer, and found a substantial discrepancy between the two. Fourier analysis suggests that the non-uniform arrangement of the melanosomes within the barbule is responsible for this discrepancy by creating variation in the periodicity. Our results suggest that a simple model in which the parameters for internal structures are averaged cannot fully explain the variation in the structural colors observed in biological samples such as the feathers of birds.

## Introduction

A number of bird species have evolved vivid plumage colors that are used as signals for various purposes [1]. Plumage colors in birds have attracted significant interest from researchers who investigate naturally occurring color production mechanisms [2, 3]. Coloration in birds can be categorized into two primary mechanisms: light absorption and light scattering. In light absorption-based coloration, pigments such as melanin, carotenoid, and psittacofulvin absorb certain wavelengths and reflect others depending on their molecular components and overall structure [4], producing matt and non-iridescent colors of unique hues. The wavelength range of absorption are dependent on the molecular structure of the pigment. When the frequency of the light matches the eigenfrequency of the pigment, the light is absorbed [4]. In contrast, light scattering produces structural color in which a periodic change in the refractive indices of the internal nanostructures leads to the strong scattering of certain wavelengths [5–8]. When the reflected waves produced by the scattering are in phase, constructive interference occurs, while destructive interference occurs when they are out of phase. Structural colors can be iridescent if the reflectance spectrum is highly dependent on the orientation of the internal nanostructure. If the shape and/or arrangement of internal nanostructure is anisotropic, the periodicity that the traveling light experiences through the internal structure varies depending on the incident angle. Thus, the pattern of light scattering and the outcome of interference change with the incident angle and iridescence, the change of light properties depending on the viewing angle, is observed [9–12].

Numerous studies have been conducted in order to understand the variety of color-producing mechanisms and the contribution of anatomical elements to the structural coloration of bird plumage. A bird feather consists of a rachis (the main shaft), barbs (second-level branches emerging from the rachis), and barbules (third-level branches emerging from the barbs) [13]. Two types of barbules are present: proximal and distal barbules, which run under and over the adjacent barb, respectively. It has been reported that, in many cases, non-iridescent structural colors are produced by the barbs while iridescent colors are produced by the distal barbules [14]. Barbs and barbules have similar constituents (i.e., β-keratin, melanosomes, and air) but their nanostructures differ. In barbs, quasi-ordered sphere-shaped or noodle-shaped air holes are distributed within the β-keratin matrix [12]. In this case, the incident light is scattered at the boundary between the air and β-keratin. The nanostructures do not have a specific orientation, thus the scattered light does not vary [12] or only slightly varies [15] with the observation angle. In contrast, melanosomes, which can have various shapes (e.g., plate-shaped, cylindrical, or air-filled cylindrical), are embedded within the β-keratin matrix of the barbules. In particular, cylindrical melanosomes usually form hexagonal or square arrays within the β-keratin matrix [12], and light scattering occurs at the boundaries between the β-keratin and melanosomes or the interface of the air and the melanin within a melanosome. This scattered light can also create interference [12].

The crow family (*Corvidae*) is one of the model systems for understanding the evolution of structural coloration in plumage [14], particularly magpies, including Eurasian, Oriental, American, and yellow-billed magpies (*Pica pica*, *P*. *serica*, *P*. *hudsonia*, and *P*. *nuttalli*). These species are distributed over a wide range of temperate and subtropical regions and exhibit iridescent colors in their tail and wing feathers, ranging from green to navy and violet. The structural colors in the feathers appear as a mosaic of different colors under a microscope [16], but they are perceived by the naked eye as distinct single hue [17].

The internal structures of the feathers have been analyzed to understand the color production mechanisms. Iridescent structures that contain β-keratin, melanin, and air holes have been classified by Durrer and redrawn by Prum [12]. The barbules of magpies are type 5, in which the multi-layered hollow melanosome cylinders are embedded in the β-keratin matrix [18]. Layers of β-keratin, melanin, and air inside hollow melanosomes can be considered a form of thin film, and numerical analysis and experimental reproduction of the optical properties arising from the thin-film effect created by the internal structures of the barbules in magpie tail feathers have been successfully conducted [16, 19]. However, these previous studies have relied on average values for the parameters associated with these nanostructural arrangements, and it is unclear whether the actual structures and their associated irregularities can predict structural colors. In many biological organisms, micro- and nanostructures often do not conform to well-defined photonic crystal structures but contain some degree of irregularity. For example, in most optical modeling where two-dimensional structures are considered, melanosomes are considered to be arranged as a hexagonal array with a constant periodic length. However, this is not a realistic representation of the internal structure because the distribution of melanosomes may vary within a barbule [12]. This creates inherent variation in the periodicity of the nanostructure of the melanosomes, which has been largely overlooked in optical studies. So far there have been a couple of studies where unmodified images that contain natural variation in the nanostructures were used for the analysis [20, 21], but the effect of the variation was not addressed. Thus, it needs to be determined whether the variation or irregularity inherently present in the internal structures of biological organisms influences the prediction of structural color.

In this report, we attempted to predict the reflectance spectra using finite-difference time-domain (FDTD) simulations based on the nanostructures derived from actual transmission electron microscopy (TEM) images of the barbules rather than an averaged model. We focused on green barbules because green is the dominant color in the tail feathers of magpies, covering more than three-quarters of the tail area. The simulated reflectance spectra were then compared with those measured using a spectrometer.

## Materials and methods

### Sample information

We collected freshly molted tail feathers from the Oriental magpie (*P*. *serica*) on the campus of Seoul National University, which hosts a stable breeding population of ~40 breeding pairs. The feathers were gently washed in 70% EtOH followed by distilled water before being air-dried and stored in a desiccator.

### Optical microscopy and spectrometry

Barbs taken from the tail feathers were observed with an optical microscope (Nikon ECLIPSE LV-N) equipped with a halogen light source. Nikon 10x (NA 0.25) and 100x (NA 0.90) objective lenses and a Nikon 10x (NA 22) ocular lens were used. The reflectance spectra were recorded using a CCD spectrometer (SM245; KSP). A halogen OSL1 light source with liquid light guides (Thorlabs) attached was used. The source from the light guides was set to be less than 10 degrees from normal, and the spot size used to illuminate the target was 0.85 mm. For the reference spectra, we used a white diffuse reflectance standard (Labsphere). We obtained the reflectance spectra from the middle area of the green region in the uppermost tail feather of the Oriental magpie.

### Electron microscopy

The samples were investigated using a scanning electron microscope (SEM; JSM-7401F; JEOL) operating at 5 kV. Before SEM analysis, the strand of the barb was coated with Pt for 50 sec at 40 mA using a sputter coater (208 HR; Cressington) and mounted on stubs with carbon tabs.

For TEM analysis, we prepared a resin block for the uppermost tail feather, which was colored dark green. We cut the feather into 5 × 5 mm pieces, which were fixed in 2.5% glutaraldehyde for 4 h at 4℃. After washing with 0.05 M sodium cacodylate buffer three times, the feather pieces were placed in 1% osmium tetroxide for 2 h at 4℃ for post-fixation staining, after which they were rinsed with distilled water three times. Serial dehydration in dry ethanol (30%, 50%, 70%, 80%, 90%, and three times at 100%) was subsequently conducted. The feather pieces were immersed in propylene oxide twice for 10 min, and then resin infiltration was conducted with the mixture of 1:1 propylene oxide and Spurr’s low viscosity resin for 4−8 h, followed by 100% Spurr’s resin for 4 h. The 100% resin was replaced twice while the samples were stored under a vacuum. The samples were then placed in molds and the blocks were cured in an oven at 60℃ for 48 h. Ultra-thin sections (65−70 nm) were produced using an ultramicrotome (Leica EM UC7, Germany) equipped with a diamond knife (Diatome, Switzerland). The sections were collected with 200 mesh copper grids and observed using a transmission electron microscope (FEI Talos L120C, Czech) under an accelerating voltage of 120 kV at magnifications of 600–10,000.

### Image processing and FDTD simulations

The cross-sections of the distal barbules were examined using TEM to determine the internal nanostructures responsible for iridescence. When we sampled the barbules, we randomly chose the tail feather but consistently used the green colored region of the feather. Based on the TEM images, we conducted FDTD simulations with MEEP software and calculated the reflectance spectra for the magpie’s distal barbules [22, 23]. Briefly, FDTD simulation proceeds by dividing the space into small grids, and the electric fields and magnetic fields in the grids are sequentially calculated using Maxwell’s equation with time flow [24]. From this calculation, one can simulate the effects of scattering, transmission, reflection and absorption of light in a given structure. In the FDTD simulations, we approximated the air holes in the barbules as circles and numerically extracted their radii and centers from TEM images of barbules using the image analysis module in MATLAB (see S1 Fig for schematic of the inner nanostructure). However, because the air holes in the TEM images were not perfect circles (S2A Fig), there were some discrepancies between the extracted circles and the air holes in the TEM images S2B Fig. These discrepancies were corrected by optimizing the pixel fraction of air holes; after calculating the pixels for the air holes (the white area) in relation to those for the keratin region (the black area), we adjusted the radii of the air holes to produce a difference that was smaller than 5% of the pixels of the air holes. The thickness of the melanosome shell was obtained from 10 randomly chosen melanosomes from each TEM image and averaged. We then set the centers of the melanosomes to be the same as that of the air holes and assigned refractive indices of 1.55 and 1 to β-keratin [25] and the air holes, respectively. We visually confirmed that the processed final image was congruent with the original image. The preparatory steps for FDTD simulations are summarized in S2 Fig. We conducted FDTD simulations using four TEM images (S3 Fig) and four sections from a single TEM image (S4 Fig).

For the FDTD simulations, the mesh size of the image was set at 0.01 μm, and normal incidence light from a Gaussian source was assumed:

$$J(\omega )\equiv {\frac{1}{\Delta \omega }e}^{i\omega t_{0}-\frac{{\left(\omega -\omega_{0}\right)}^{2}}{2{\Delta \omega }^{2}}}$$ (1)

where J(ω) is the current and *ω*_0_/2*π* and Δω/2π are the center frequency and width 1/500 nm^-1^ and 1/250 nm^-1^, respectively. Although we used a continuous source for the experiment, Gaussian source was assumed in the simulation for the efficiency. Due to the natural shape of the internal structure, the light incidence to the structure in our FDTD is not normal but rather in an oblique manner. FDTD on the simulation of normal incidence light propagated into oblique photonic nanostructure has already been conducted by a previous study [20] in which the simulated and observed spectra well agreed. The polarization of the propagating waves can be either transverse electric (TE) or transverse magnetic (TM) mode. In TE mode, the electric field vector is perpendicular to the long axis of the cylinder while, in TM mode, the electric field is parallel to the long axis of the cylinder. We used both modes to obtain the reflectance spectra because the white light used in the experiment was un-polarized. We used the refractive index for melanin determined by Stavenga et al. [25], and the dispersion was fit with a Lorentzian function (see Supplementary Materials for details). The simulated cell size is depicted in S5 Fig. The prepared object was located at the center of the simulation cell. The width of the cell, the height, and the depth were about 10 μm, 5 μm, and infinite, respectively. The width and height of the cell varied depending on the size of the TEM image. We set the depth to be infinite because the length of the melanosome was much bigger than its radius (Fig 2B in ref [26]). At the end of the x-direction, there was the perfectly matched layer (PML), which is artificial absorbing later for the wave equations, and the periodic boundary condition (PBC) is set at the end of the y-direction. The thickness of PML was about 700 nm (about the wavelength of incident lights). The source and the detector (the red and the blue lines) were located in front of the PML (S5 Fig).

### Fourier analysis

Because the light transmitted through a structure is reflected at the interface where the refractive index changes, the reflectance spectra of the structure can be expected to be associated with the periodicity of the refractive index array. Thus, we conducted Fourier analysis of TEM images, in which the refractive index is expressed as contrast, to examine the periodicity of the internal structures of the barbules.

We randomly selected four sections of a single-barbule TEM image (~ 1 x 1 μm^2^ each, corresponding to the size of the color domains) used in the FDTD simulations. We hypothesized the color observed from the barbule is produced by the summation of the color produced at each domain. Thus, by setting the size of the section close to the domain size, we aim to simulate the presence of various colors of visible at each domain. Each section was Fourier transformed using the FFT function in MATLAB. The Fourier-transformed images exhibited the periodicity of each section in the spatial frequency *K* (*nm*^−1^), which was difficult to identify in the original TEM images. Because the structural color of the feathers was investigated using normal incident light, only the lateral axis in the Fourier-transformed images was considered. The expected reflectance wavelength λ_r_ was calculated using Eq (2):

$$\lambda_{r}=\frac{2\pi n_{eff}}{K_{p}}$$ (2)

where *n*_*eff*_ and *K*_*p*_ are the effective refractive index and the peak point of Fourier power spectrum, respectively, in Fourier space for each section. We implemented the effective refractive index, which calculated using filling fraction (S6 Fig). We drew the histogram of the magnitude of power spectrum and transformed the *K* axis to the wavelength with Eq 2. We compared the Fourier power spectra with the reflectance spectra from the FDTD simulations. We also calculated the average, standard deviation, and coefficient of variation for the internal diameter of the melanosomes and the distance between adjacent melanosomes and calculated the correlation between these structural parameters and the predicted wavelength for the peak reflectance.

### Reflectance spectra data to RGB data

We used PAVO module in R to predict RGB values from the reflectance spectra obtained from FDTD simulations. These values were compared with the colors of the domains in the barbule.

## Results

**Fig 1:**
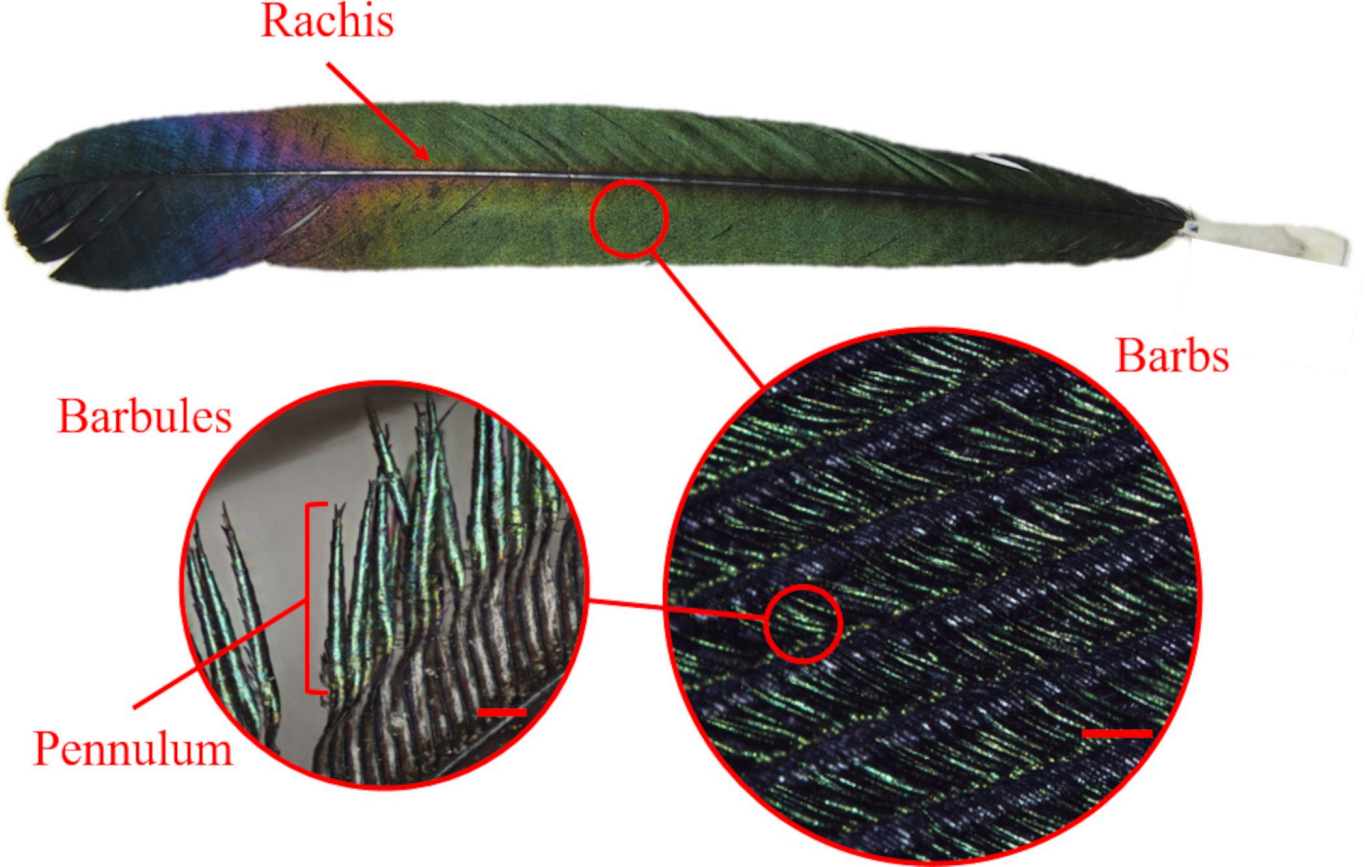
Photographs and microscopic details of the uppermost tail feather of the Oriental magpie (*P*. *serica*). The tail feather of the magpie was observed using an optical camera. The barbs extend from the rachis, and the barbules reach out from the barbs in succession. The surface of the flattened and elongated parts (pennulum) of the distal barbules is iridescent green, transforming into iridescent violet and navy around the tip of the feather. The scale bars in left and right represent 50 μm, 200 μm, respectively.

**Fig 2:**
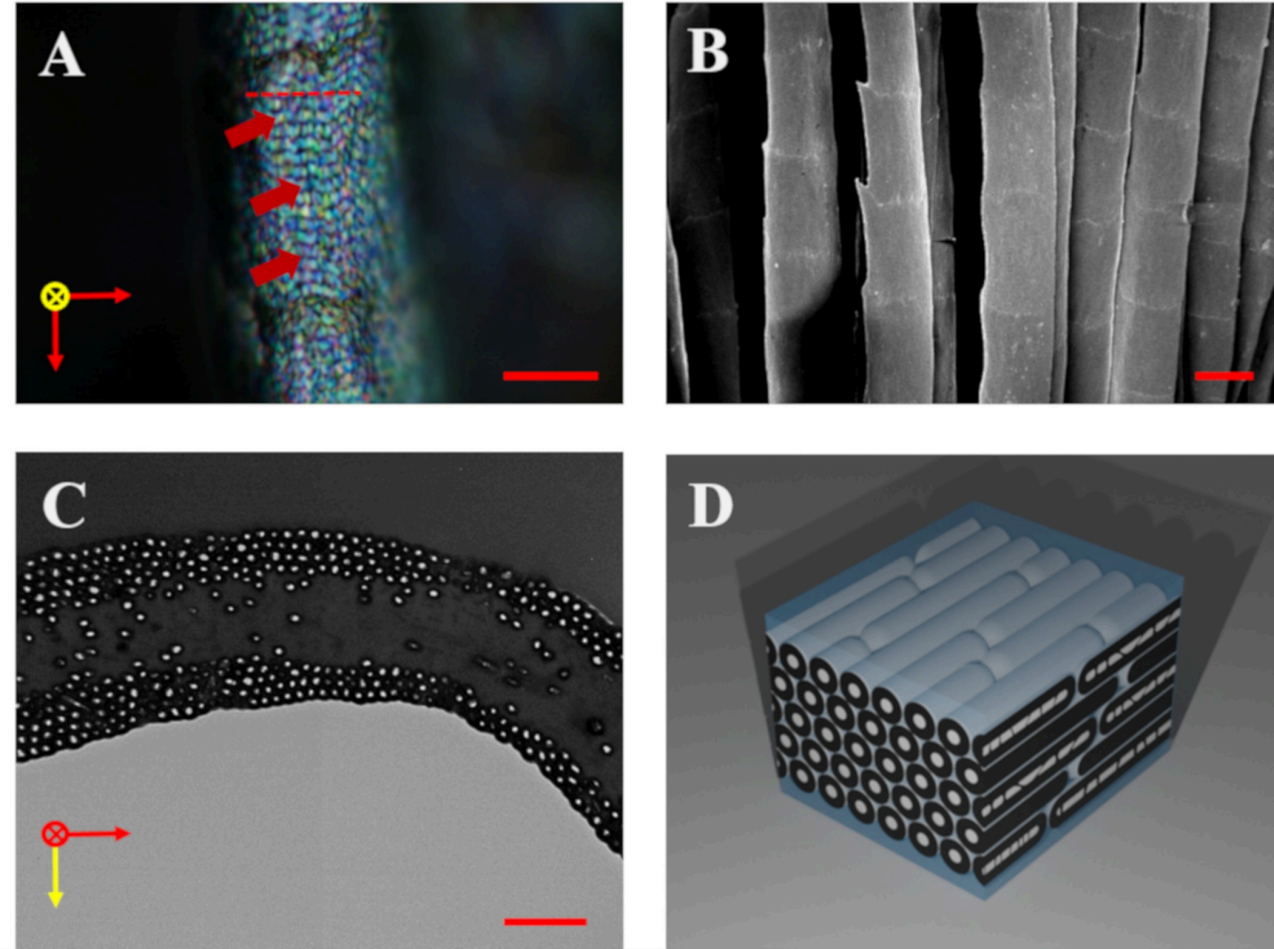
Optical and electron microscopic images and the structural model of the barbule. **A** Surface of the barbule under an optical microscope (x1000) showing multi-colored domains. The red arrows indicate yellow, violet, and blue from top to bottom. The dashed red line indicates the horizontal band. The yellow point represents the direction of light propagation. **B** Scanning electron microscopy image of the barbule surface. **C** Transmission electron microscopy image of the barbule cross-section showing the hexagonal structure of the melanin stack. The yellow arrow indicates the direction of light propagation. **D** Structural model of the barbule and the type of the structure classified as type 5 from Durrel’s classification. The scale bars in **A, B,** and **C** represent 10 μm, 20 μm, and 1 μm, respectively.

The tail feather of the Oriental magpie was iridescent green when observed under low magnification (Fig 1). However, under a higher magnification (x1000), a combination of blue, violet, yellow, and green iridescence was observed (Fig 2A). These color domains (marked with red arrows in Fig 2A) were arranged horizontally across the barbule (marked with red dashed lines in Fig 2A). The average width of the domains was ~1 μm. An SEM image of the surface of the pennulum exhibited no noticeable border between the domains (Fig 2B). Fig 2C presents the cross-section of the internal nanostructures in the pennulum with a height of 2 μm. These internal nanostructures consisted of β-keratin, melanin, and air holes, which appeared as gray, black, and white regions, respectively, in cross-sectional TEM images (Fig 2C). The hollow torus-shaped melanosomes were arranged in multi-layers just beneath the epicuticle. The radii of the melanosomes and air holes were approximately 84 nm and 30 nm, respectively, and the thickness of the melanosome shell was about 54 nm. The TEM images revealed that the arrangement of the melanosomes in the nanostructures was hexagonal on average (as modeled in Fig 2D), but the internal layers of melanosomes deviated from this hexagonal arrangement (Fig 2C).

**Fig 3:**
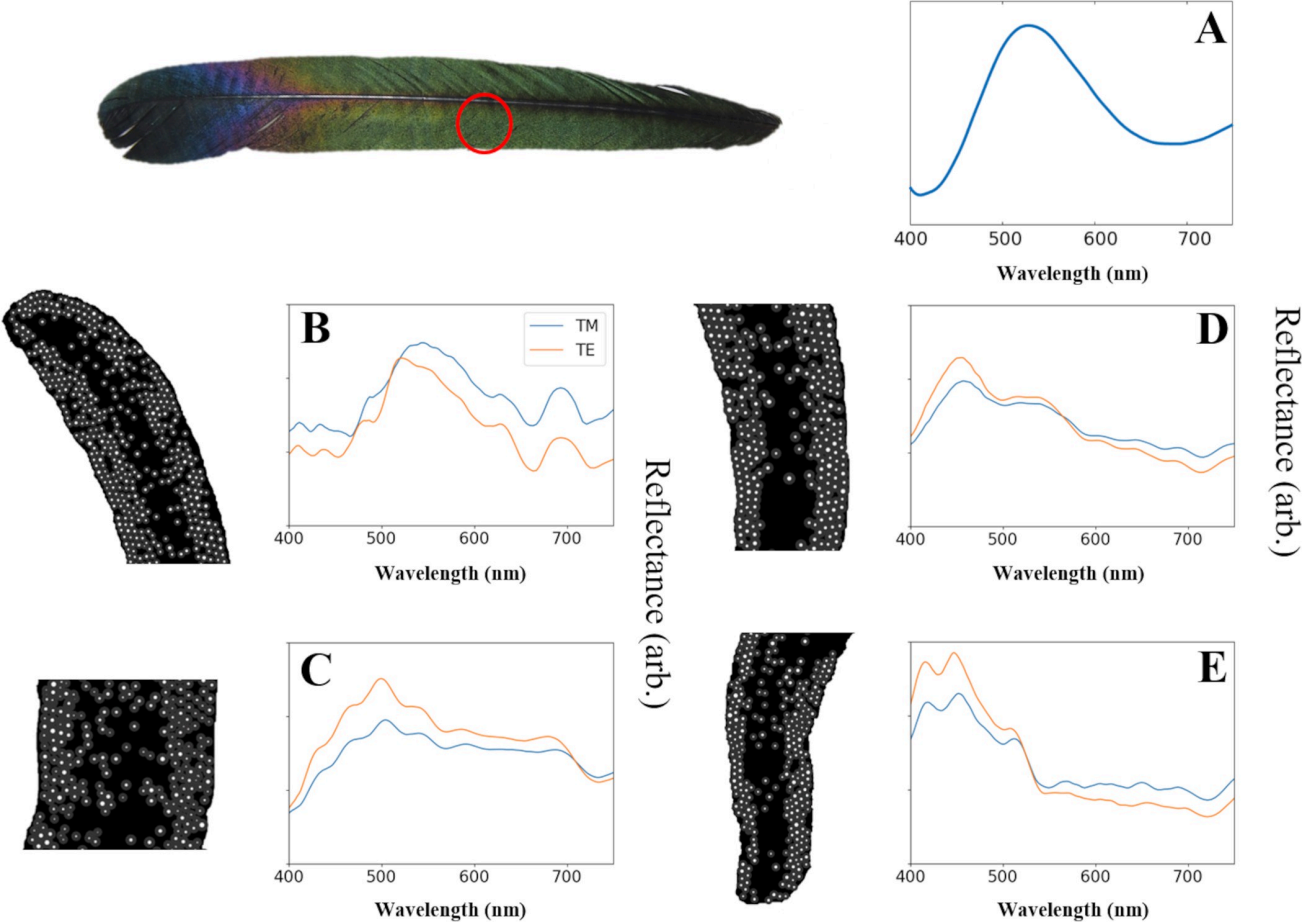
Reflectance spectra measured using the experimental setup and calculated using FDTD simulations. **A** Reflectance spectra of the barbule in the green area indicated by the red circle measured using the experimental setup. The wide spectrum has a dominant peak at about 520 nm. **B−E** Spectra calculated using FDTD simulations. The keratin, melanin, and air are represented by black, gray, and white in the simulated images, respectively. The refractive indices for keratin and air are 1.55 and 1.00 [25]. The refractive index of melanin was taken from Stavenga et al. [25] and fit with a Lorentzian function. The B and C spectra agree well with the experimental spectra, while the D and E spectra differ. The solid blue and orange lines represent TE and TM mode, respectively.

The reflectance spectrum for the middle of the green area of the feather had a broad peak at about 520 nm with a width of ~150 nm and was skewed to the right (Fig 3A). Generally, the spectrum agreed well with the color observed at low magnifications (Fig 1).

The FDTD results for the reflectance spectra of four barbules are summarized in Fig 3B–3E. The reflectance spectra exhibited slight differences in intensity depending on the polarization of the incident light, but the general shape of these spectra was similar (Fig 3). We focused on the general shape of the spectra rather than the slight difference in intensity. Furthermore, all the simulated reflectance spectra were skewed to the right. This is because the imaginary part of the relative permittivity of melanin has higher values at short wavelengths and exponentially decreases as the wavelength becomes longer (S7 Fig). The imaginary part of relative permittivity is absorption of melanosome and in the short wavelength it is very high so that the reflectance is decreased in the region and the gradient of the reflectance is steeper than the long wavelength region. The reflectance spectrum in Fig 3B had a broad main peak at about 530 nm under TM mode and several small peaks at the wavelengths 410, 430, 490, 630, and 690 nm. The main peak under TE mode slightly shifted toward a shorter wavelength, but the locations of small peaks were the same as for TM mode (Fig 3B). The main peak in Fig 3C was located at a wavelength of 500 nm, and four small peaks were observed at 433, 472, 535, and 625 nm with some minor peaks in the longer wavelength range (Fig 3C). The spectra in Fig 3B and 3C were congruent with the reflectance spectra obtained using the spectrometer; a broad peak with a width of ~150 nm was present within the wavelength range of 500−530 nm, while at longer wavelengths the spectra gradually decreased, with some smaller peaks.

In contrast, the spectra presented in Fig 3D and 3E differed from the measured spectrum. The reflectance spectrum in Fig 3D showed a single peak at 450 nm with wide shoulders spanning from 500 nm to 550 nm, beyond which the reflectance decreased. Fig 3E shows two dominant peaks at 415 nm and 450 nm and a third peak at 510 nm, beyond which the reflectance decreased in the long wavelength range with minor peaks.

The major difference between the four simulated reflectance spectra was the location of the main peaks. The spectra in Fig 3B and 3C had the main peak at the wavelength corresponding to green, which was similar to the measured spectra. On the other hand, the others had peaks at a shorter wavelength than the measured spectra, corresponding to blue.

**Fig 4:**
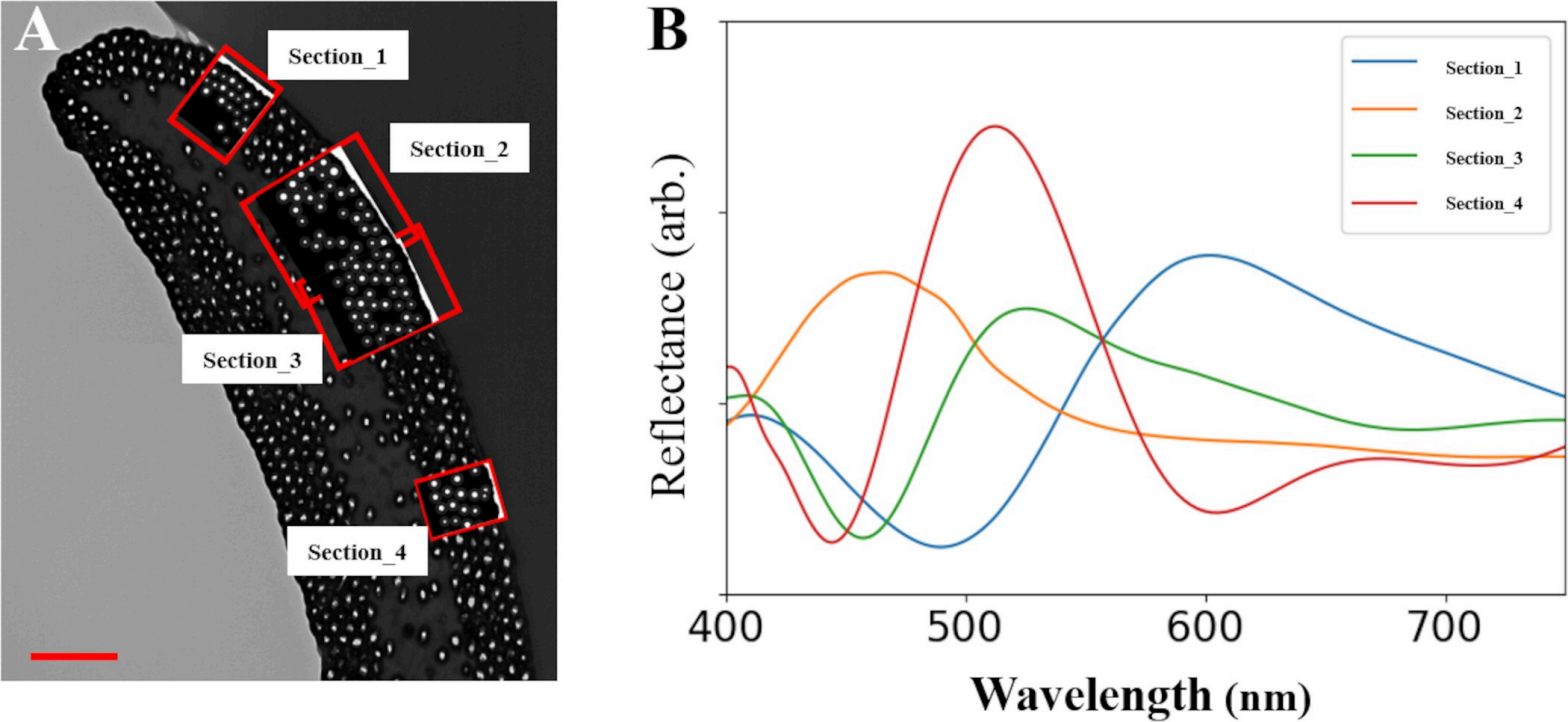
Reflectance spectra obtained from FDTD simulations. **A** Four simulated sections of the barbule, each with a height of around 1 μm, corresponding to the size of the color domain. Enlarged images are presented in S3 Fig. **B** Spectra for the sections of the barbule calculated from the FDTD simulations. Each section has different peaks. Bar (A): 1 μm.

To identify the origin of the peaks, we analyzed the internal structure of the barbule section in detail. We randomly chose four sections of a TEM image of a single barbule and conducted FDTD simulations under TE mode (Fig 4). We only used TE mode here because the difference in the main peaks between TE and TM modes was minor (Fig 3). The length of the barbule section used for the simulations was ~1 µm, corresponding to the size of the color domain. The peak wavelengths predicted in the FDTD simulations for each section were different and they deviated from the main peak at 520 nm measured from the feather (Fig 4). The reflectance spectra were converted to RGB data by PAVO module in R. The colors from the RGB data had various colors (red, blue, yellow and green) as shown in the domains in the barbule’s surface (S8 Fig). Even though the sections did not exactly represent a single domain, it was observed that the structural color varied within the barbule. In addition, it can be noted that the spectra obtained from the four sections do not contain any ripples (Fig 4) but those from the barbules do (Fig 3). This indicates that the ripples in Fig 3 is not an outcome of numerical errors in our FDTD simulations, but rather an outcome of summation of spectra observed at the size of sections we used (Fig 4).

**Fig 5:**
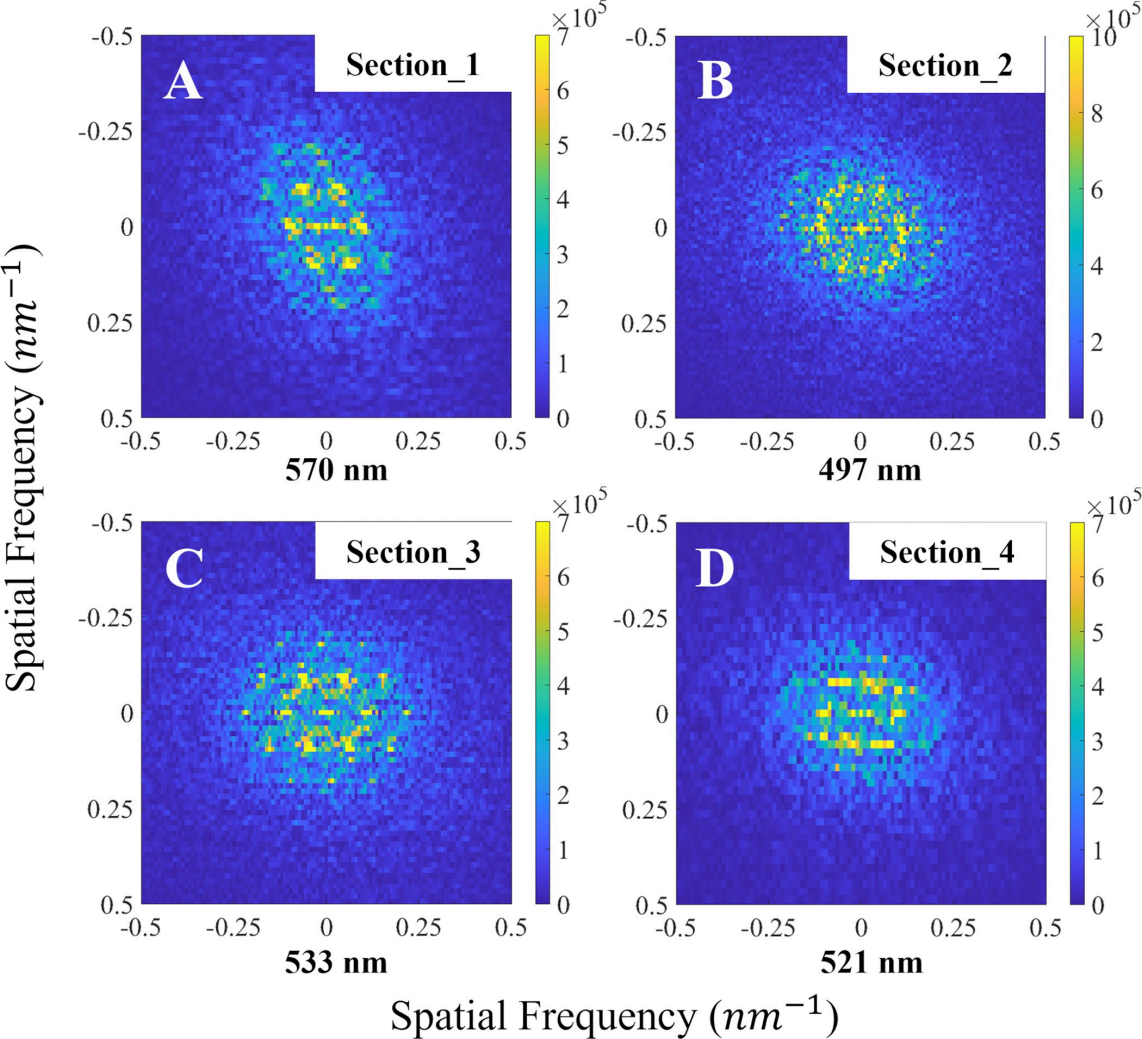
Fourier transforms for the sections from a single-barbule TEM image. The Fourier transforms for the refractive index distribution in the barbule are expressed in spatial frequency. The peak wavelengths predicted from Fourier analysis are listed under each image.

We also conducted Fourier analysis on the four sections of a TEM image of a single barbule. Fourier transforms of all four sections expressed periodicity in terms of the spatial frequency *K* (*nm*^−1^). The peaks in the Fourier transforms indicated the presence of hexagonal arrangements of melanosomes in sections 1 and 3 (Fig 5A, 5C), which matches the type 5 melanosome structure shown in Fig 2D. However, the predicted peak wavelengths for sections 1 and 3 were quite different (570 nm and 533 nm, respectively). A hexagonal arrangement was not observed in sections 2 and 4 (Fig 5B and 5D), although some periodicity was visible. The reflectance spectra of sections 3 and 4 were similar (peaks at 533 nm and 521 nm, respectively) and these were also similar to the spectra recorded for the whole feather (peak at ~ 520 nm). In addition, the Fourier power spectra and reflectance spectra from the FDTD simulations closely matched (S9 Fig). Of the structural parameters that we considered, the average distance between the adjacent melanosomes was most strongly correlated with the wavelength of the peak reflectance (Table 1).

**Table 1:** Relationship between the structural parameters and the wavelength of the peak reflectance predicted from Fourier transform analysis.

Sections	Image analysis										Fourier transform analysis		
	Thickness of cortex			Internal diameter of melanosomes			Distance between adjacent melanosomes			r/a	Hexagonal array	Peak reflectance (nm)	Refractive index
	(nm)			(*r*, nm)			(a, nm)						
	Av.	SD	CV	Av.	SD	CV	Av.	SD	CV				
1	20.20	2.70	0.13	27.67	9.05	0.33	348.7	3.41	0.01	0.079	Yes	570	1.635
2	27.10	3.98	0.15	25.77	7.72	0.3	303.2	5.63	0.019	0.085	No	498	1.638
3	27.80	2.97	0.11	23.53	5.88	0.25	325	1.89	0.006	0.072	Yes	533	1.643
4	34.40	5.54	0.16	30.73	7.42	0.24	320	5.03	0.016	0.096	No	521	1.628
Correlation coefficient with peak reflectance wavelength	-	-	-	0.081	0.432	0.407	0.998	-0.601	-0.681	-0.408	-	-	-

From the TEM images of four sections, the average, standard deviation, and coefficient of variation for the internal diameter of the melanosomes (*r*) and the distance between adjacent melanosomes (lattice constant, *a*) were calculated. We also calculated the correlation coefficient between the structural parameters and the wavelength of the peak reflectance and found that the average distance between adjacent melanosomes greatly contributed to this wavelength.

## Discussion

When observed under an optical microscope, the surface colors of the barbules of the green area of the magpie feather contain blue, violet, and yellow in addition to green. This variation in the surface color was reported by a previous study [16], but it has been ignored in previous simulations based on simplified models. In particular, simplified arrays of melanosomes representing the barbule structure have been employed in simulations, leading to simplified reflectance spectra, many with a single distinct peak that leads to the simple prediction of color [16, 18]. This is because the refined nanostructure simplifies the periodicity of the structure, but the internal nanostructures in real barbules are not uniform in size and distribution. Though the causes for this variation in the internal structures are not well known, it is known that cylinder-like melanosomes in type 5 barbules are not precise cylinders with truncated ends; rather, they have an elongated ellipsoidal shape with rounded ends. This inherently leads to variation and irregularities in the size of and distance between melanosomes, especially at their tips. Even small differences in the radius or density of the melanosomes can alter the interaction between the light and the structure, thus affecting the reflectance wavelength [16, 18, 19]. How the irregularities in the size and distribution of melanosomes arise remains unclear. If the longitudinal shape of the melanosomes is the main source of these irregularities, then longer melanosomes should have less variation in their structural color. In order to validate this hypothesis, longitudinal sections of the colored regions of feathers should be examined.

Our results from FDTD simulations using raw TEM images confirmed the presence of irregularities that affected the reflectance wavelength. Because we did not simplify the structures, the reflectance spectra had numerous minor peaks in addition to the main peak at ~520 nm that matched the peak from the measured spectrum (Fig 3B and 3C) or had main peaks located in the shorter wavelength regions (Fig 3D and 3E). These results differ from those from previous studies that employed ideal models that produced a strong single peak, representing a single color [16, 18].

In addition, the FDTD simulations conducted on four randomly selected sections of a TEM image of a single barbule predicted the presence of different main peaks although the spectra contained simpler shapes than predicted from the TEM image for the entire barbule. The results indicate that each section of the barbule had different reflectance wavelengths, which may explain the presence of color domains observed under the light microscope. In other words, the irregularity in the barbule could be responsible for the production of various surface colors.

Results from Fourier analysis conducted on four sections of a single barbule also suggested that the presence of irregularities in the melanosome structure and arrangements (the Fourier transform of simple hexagonal array was shown in previous study [27]) that led to the deviation from a simple hexagonal arrangement may be responsible for the variation in the structural color even within a single barbule by changing the periodic length or radii of the melanosomes. In previous studies [28, 29], it was suggested that the cortex layer could also contribute to barbule’s structural color, but the cortex of magpie barbule was too thin to significantly influence the barbule’s color (163.4±1.8 nm, 275±30 nm in refs. [28, 29] and 27.5±7.5 nm in our samples; Table 1). The importance of irregularities in the color production is supported by the comparisons between sections 1 and 3; although both sections contained hexagonal arrangements of melanosomes, the locations of the predicted spectral peaks were considerably different (570 nm and 533 nm, respectively). Thus, a slight deviation in the regular hexagonal arrangement can cause a substantial change in the reflectance spectra. Furthermore, the similarity of the reflectance spectra for sections 3 and 4 (peaks at 533 nm and 521 nm, respectively) and their similarity with the spectra measured from the entire feather (peak at ~ 520 nm) indicated that the presence of a hexagonal arrangement itself was not responsible for the lattice constant (i.e., periodicity) of the nanostructure (see *a* in Table 1), which the Fourier analysis demonstrated was mainly responsible for the green color observed on a broader scale. The comparison between the lattice constant and peak reflectance revealed that a higher lattice constant led to a longer peak wavelength (Table 1). After the main peaks were approximated, the effective index of the barbule, which had the same trend in the ratio of the lattice constant to the melanosome radius as that calculated from each TEM image (*r/a* in Table 1), created a small deviation from the main reflectance peaks.

Based on Fourier transformation (Eq 2), longer periodic lengths (i.e., a smaller *K*) were predicted to shift the reflectance spectra to longer wavelengths. Furthermore, although we could not precisely predict how the reflectance spectra would change according to the variation in the filling fraction of the melanosome using Fourier analysis, we predict that, when the average periodicity of the structures are similar, thicker melanosomes walls will shift the reflectance spectra to longer wavelengths. Overall, our results suggest that the variation in the size and arrangement of feather nanostructures (i.e., melanosomes in magpies) contribute to the variation in the color observed under the microscope and predicted from FDTD simulations and Fourier analyses. Thus, due to the irregularities in internal structure, the barbules of the Oriental magpie tail feathers can be concluded to have quasi-ordered two-dimensional nanostructures.

In summary, we investigated the relationship between the irregularities in the internal structures and the resultant structural color in the tail feathers of the Oriental magpie and found that the irregularities and variation in the internal structures created variation in the structural color by changing the periodic length. Our results suggest that a simple model with average values for internal structures and their arrangement cannot fully represent the local structural color information in biological samples such as the feathers of birds. In future research, it needs to be clarified how irregularities in the radii of and distances between melanosomes contribute to the difference in the reflectance spectra.

## Supporting information

S1 Fig Schematic of nanostructure in the barbule. The inside of the barbule consist of melanosome, which has hexagonal array. The inner radius of melanin cylinder r and the periodic length a are depicted as black and red arrow, respectively.
(TIF)

S2 Fig Preparatory steps for FDTD simulation. **A** TEM image of a distal barbule exhibiting green iridescence. The melanosomes are not uniform in size and the image contains irregularities. **B** Extracting the location and radius of the melanosomes. The air hole radius was determined by assuming a melanosomes shell thickness of 53 nm. **C** Setting the refractive indices of β-keratin (black area) and air (white area) at 1.55 and 1.00 respectively. The inset in **C** displays the direction of TE and TM mode polarization.
(TIF)

S3 Fig A−D TEM images of the barbules used in FDTD simulations. The area used for FDTD simulation is marked with the red dashed line. All images are from different barbules exhibiting green iridescence.
(TIF)

S4 Fig The cut images from single barbule by sections, using for FDTD simulation. **A** TEM image used for FDTD simulations with the red dashed box representing the section used for the simulation. **B-E** FDTD simulation images of the red dashed box. Arrangements and the sizes of melanosomes vary even in the single barbule.
(TIF)

S5 Fig Schematic of FDTD simulation. The prepared geometry for the simulation is located at the center of the simulation area. PMLs are located at each end of the x-direction and at each end of the y-direction, there are periodic boundary conditions. The source and the detector are represented by red and blue lines, respectively.
(TIF)

S6 Fig The cut TEM image and the MATLAB image for calculating the pixels. **A** The cut TEM image of the section_3 in Fig 4. **B** The image transformed using MATLAB. The air-holes and melanin are represented by yellow and mint green, respectively.
(DOCX)

S7 FigRefitted relative permittivity of melanin with a Lorentzian function.**A** Real part of the relative permittivity of melanin. **B** Imaginary part of the relative permittivity of melanin. The real part of $\sqrt{\varepsilon_{r}}$ represents the refractive index of melanin and the imaginary part of ε_*r*_ represents the absorption of melanin. The blue and orange curves in both plots represent the reference determined by Stavenga et al. [25] and the refitted curve via a Lorentzian function.(DOCX)

S8 Fig The reflectance spectra data and RGB data obtained from the four sections in S4 Fig. The colors from RGB data are represented in the background colors, and the RGB values are written on the background.
(TIF)

S9 Fig A−D Fourier power spectra (blue bars) and the reflectance spectra (orange solid lines) obtained from FDTD simulations of the sections in S3 Fig. The expected spectra from Fourier analysis agree closely with the reflectance spectra.
(DOCX)
