# Supplementary figures and images for: Effect of nanostructural irregularities on structural color in the tail feathers of the Oriental magpie Pica serica
Source: PLoS One. 2023 Mar 22;18(3):e0282053. doi: 10.1371/journal.pone.0282053 (PMC10032483; doi:10.1371/journal.pone.0282053)

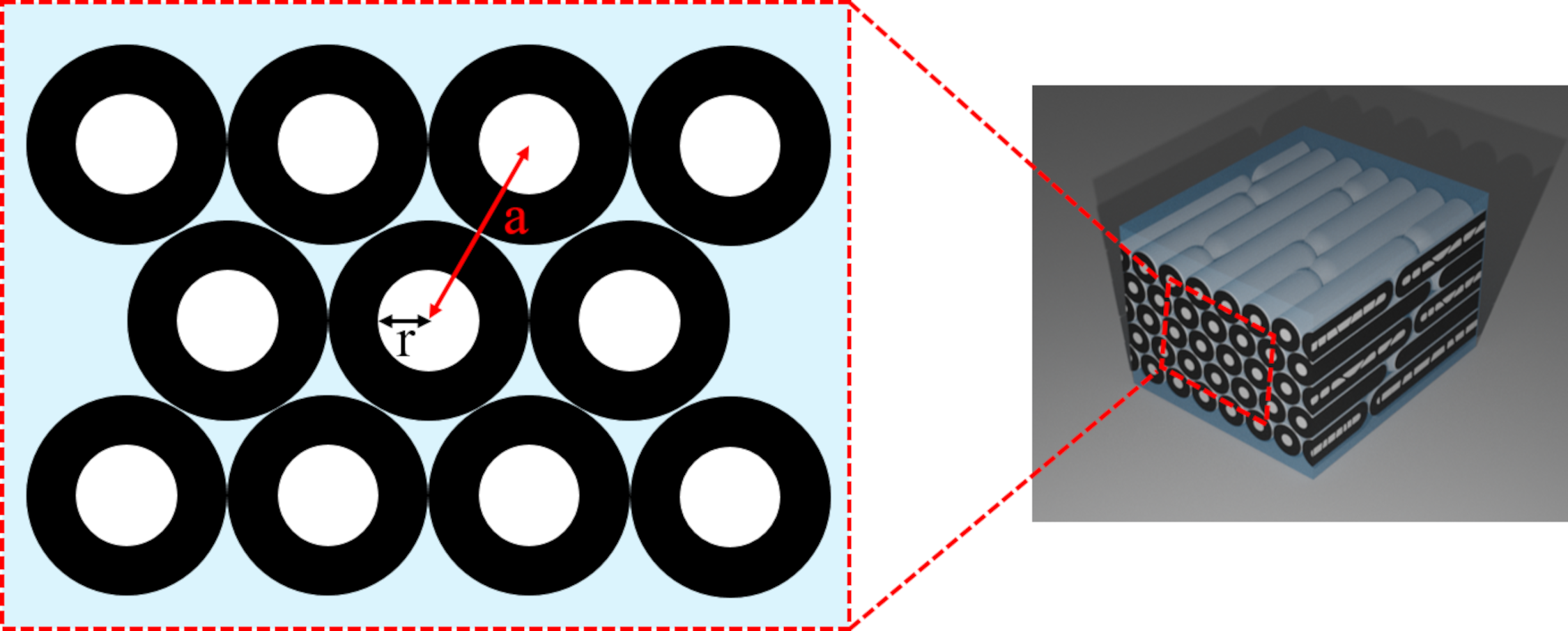

Supplement: S1 Fig — The inside of the barbule consist of melanosome, which has hexagonal array. The inner radius of melanin cylinder r and the periodic length a are depicted as black and red arrow, respectively. (TIF) [file pone.0282053.s001.tif]

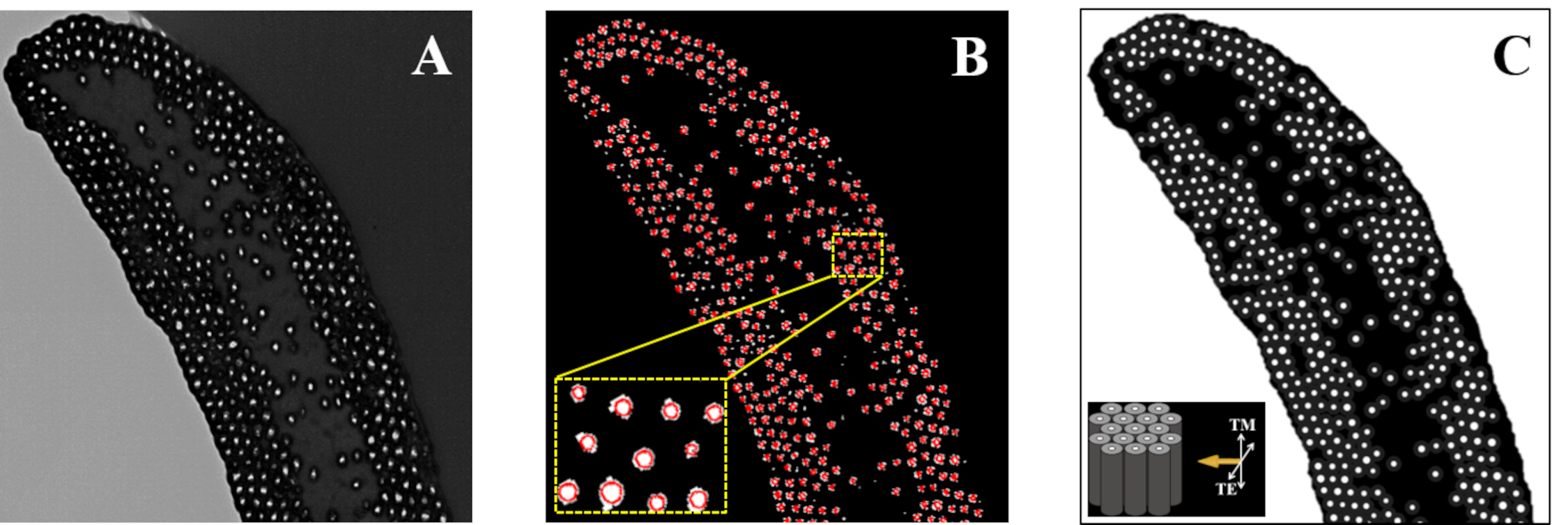

Supplement: S2 Fig — A TEM image of a distal barbule exhibiting green iridescence. The melanosomes are not uniform in size and the image contains irregularities. B Extracting the location and radius of the melanosomes. The air hole radius was determined by assuming a melanosomes shell thickness of 53 nm. C Setting the refractive indices of β-keratin (black area) and air (white area) at 1.55 and 1.00 respectively. The inset in C displays the direction of TE and TM mode polarization. (TIF) [file pone.0282053.s002.tif]

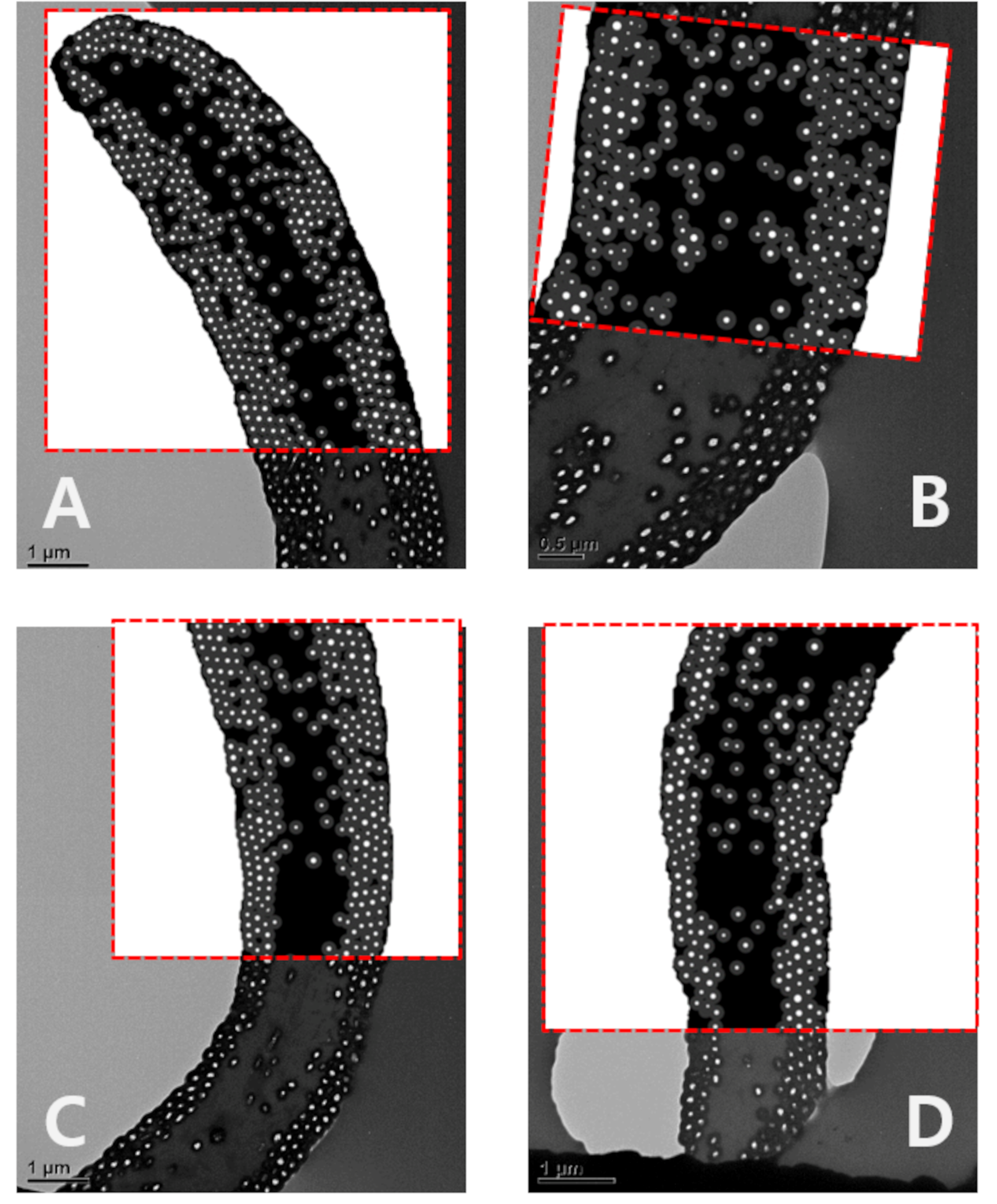

Supplement: S3 Fig — The area used for FDTD simulation is marked with the red dashed line. All images are from different barbules exhibiting green iridescence. (TIF) [file pone.0282053.s003.tif]

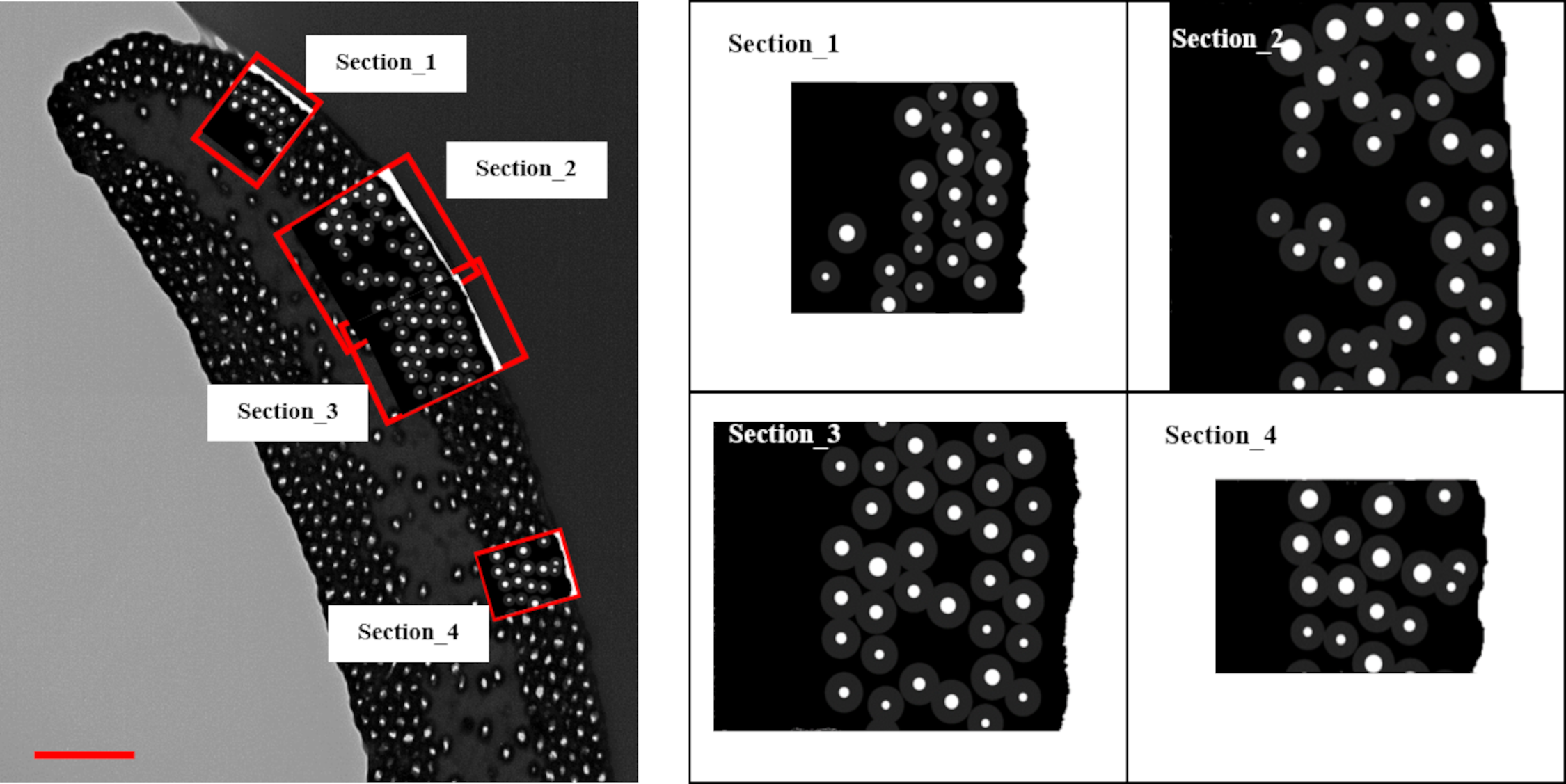

Supplement: S4 Fig — A TEM image used for FDTD simulations with the red dashed box representing the section used for the simulation. B-E FDTD simulation images of the red dashed box. Arrangements and the sizes of melanosomes vary even in the single barbule. (TIF) [file pone.0282053.s004.tif]

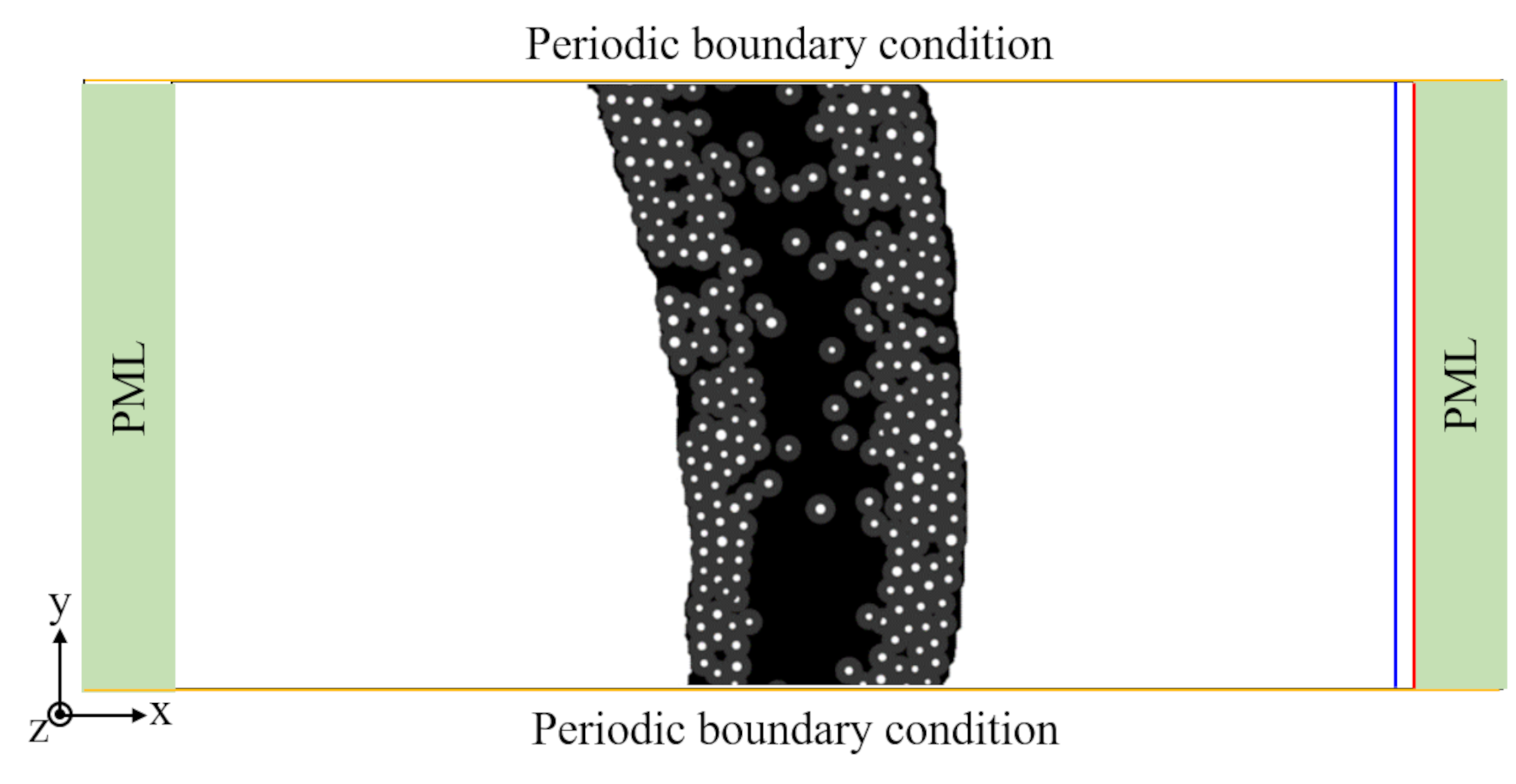

Supplement: S5 Fig — The prepared geometry for the simulation is located at the center of the simulation area. PMLs are located at each end of the x-direction and at each end of the y-direction, there are periodic boundary conditions. The source and the detector are represented by red and blue lines, respectively. (TIF) [file pone.0282053.s005.tif]

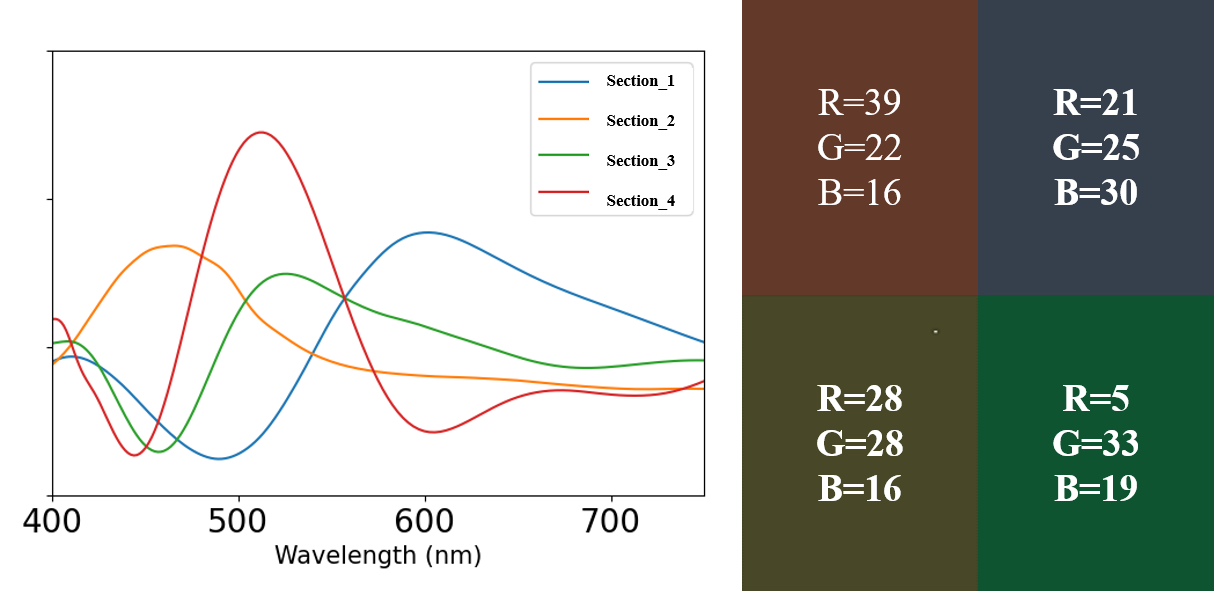

Supplement: S8 Fig — The colors from RGB data are represented in the background colors, and the RGB values are written on the background. (TIF) [file pone.0282053.s008.tif]
