# Supplementary material for: Effect of nanostructural irregularities on structural color in the tail feathers of the Oriental magpie Pica serica
Source: PLoS One. 2023 Mar 22;18(3):e0282053. doi: 10.1371/journal.pone.0282053 (PMC10032483; doi:10.1371/journal.pone.0282053)
Supplement: S6 Fig — A The cut TEM image of the section_3 in Fig 4. B The image transformed using MATLAB. The air-holes and melanin are represented by yellow and mint green, respectively. (DOCX) [file pone.0282053.s006.docx]

*** Setting effective refractive index**

We implemented effective refractive index to calculate the expected reflectance wavelength using Fourier transform images. The refractive index $n_{eff}$ is calculated using Eq. (3):

$n_{eff}=\sqrt{{f_{air}n_{air}^{2}+\left( 1-f_{air} \right)n}_{mel}^{2}}$ (3)

$f_{air}$, $n_{air}$, and $n_{mel}$ are filling fraction, refractive index of air and melanin, respectively. We calculated the filling fraction using cut TEM images, which we used for the FDTD simulation by section. Because of almost of the images are air-hole and melanin, we only calculated the pixels of air-holes and melanin (the background).


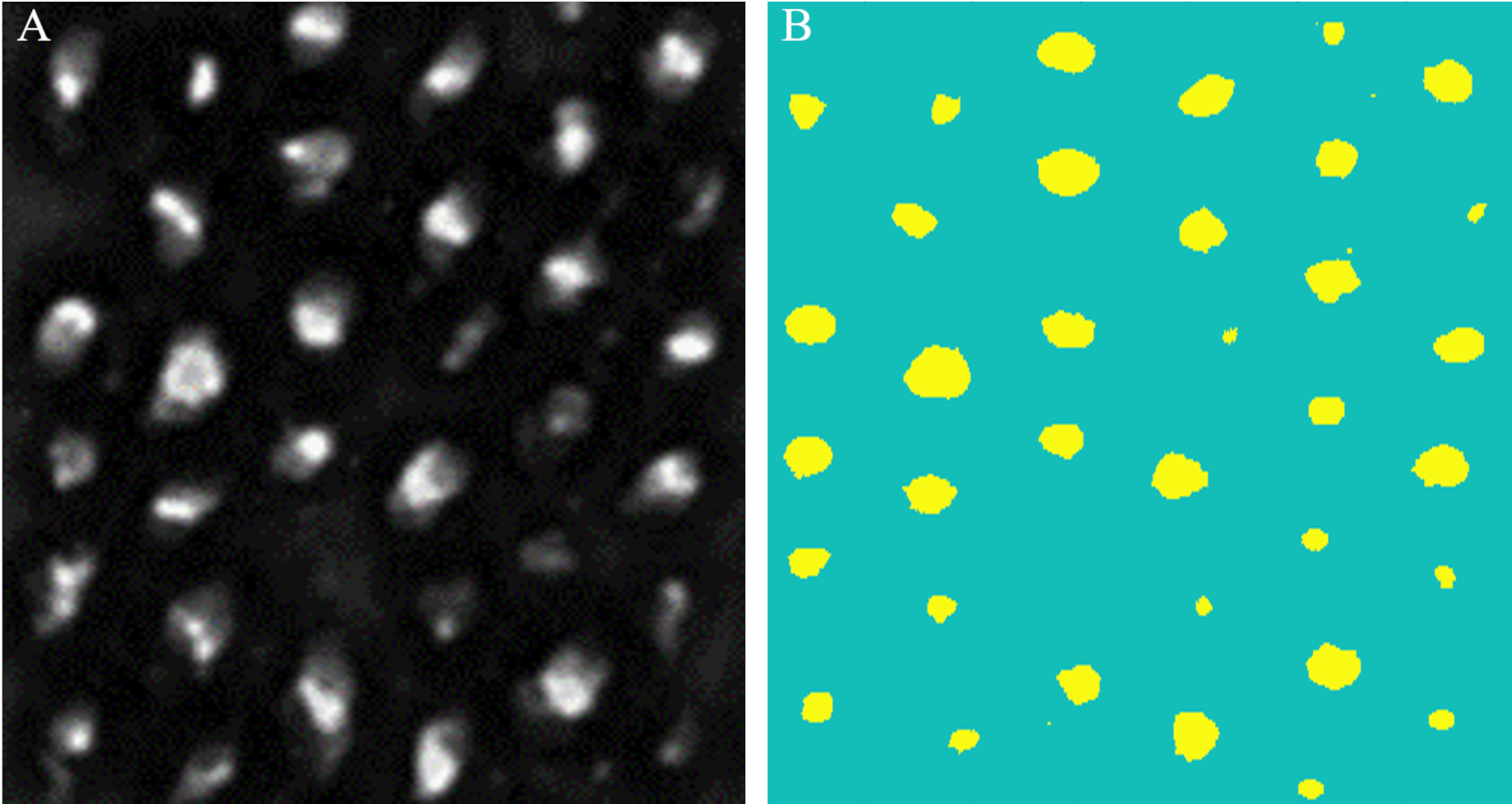


**S6 Fig. The cut TEM image and the matlab image for calculating the pixels.** **A** The cut TEM image of the section_3 in Fig 4B. **B** The image transformed using matlab. The air-holes and melanin are represented by yellow and mint green, respectively.
