# Supplementary material for: Effect of nanostructural irregularities on structural color in the tail feathers of the Oriental magpie Pica serica
Source: PLoS One. 2023 Mar 22;18(3):e0282053. doi: 10.1371/journal.pone.0282053 (PMC10032483; doi:10.1371/journal.pone.0282053)
Supplement: S7 Fig — A Real part of the relative permittivity of melanin. B Imaginary part of the relative permittivity of melanin. The real part of εr represents the refractive index of melanin and the imaginary part of εr represents the absorption of melanin. The blue and orange curves in both plots represent the reference determined by Stavenga et al. [25] and the refitted curve via a Lorentzian function. (DOCX) [file pone.0282053.s007.docx]

*** Setting the parameters for the refractive index of melanin**

Our simulation tool uses the Lorentzian distribution for dispersive materials. Therefore, we fitted the dispersion with Lorentzian function.

$\varepsilon_{r}\left( f \right)=\varepsilon_{\infty}+\sum_{n} \frac{\sigma_{n}\cdot f_{n}^{2}}{f_{n}^{2}-f^{2}-if\gamma_{n}/2\pi}$ (4)

The fit parameters were 2.22, 0.51, 0.03, and 0.05 for $\varepsilon_{\infty}$, $\sigma_{n}$, $f_{n}$ and $\gamma_{n}$ respectively. The fitted curve of the epsilon, the relative permittivity, of the melanin is depicted in S7 Fig. The blue lines are data from Ref. [25], and orange lines are fitted curves from Eq.4. We confirm the fitted curves of epsilon of melanin are well matched up with the reference curves, and use the parameters, so that the dispersion of melanin is applied for the simulations. The relative permittivity is expressed as a complex number. S7B Fig. shows that melanin has more absorption in the shorter wavelength range because the imaginary part of $\varepsilon_{r}$ represents absorption.


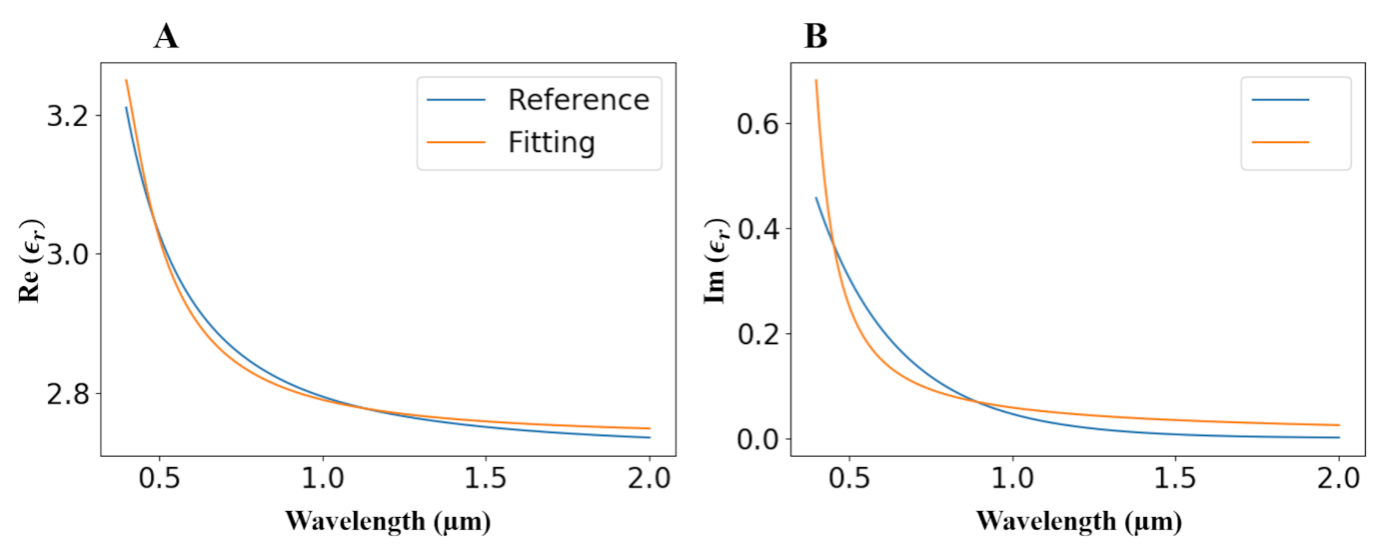
,

**S7 Fig. The refitted relative permittivity of melanin with the Lorentzian function.** **A** The real part of the relative permittivity of melanin. **B** the imaginary part of the relative permittivity of melanin. The real part of $\sqrt{\varepsilon_{r}}$ means the refractive index of the melanin and the imaginary part of $\varepsilon_{r}$ means the absorption of the melanin. The blue and orange curves on both plots represent the reference determined by Stavenga et al. [25] and the refitted curve by Lorentzian function.
