# Supplementary material for: Effect of nanostructural irregularities on structural color in the tail feathers of the Oriental magpie Pica serica
Source: PLoS One. 2023 Mar 22;18(3):e0282053. doi: 10.1371/journal.pone.0282053 (PMC10032483; doi:10.1371/journal.pone.0282053)
Supplement: S9 Fig — The expected spectra from Fourier analysis agree closely with the reflectance spectra. (DOCX) [file pone.0282053.s009.docx]

*** Comparing Fourier power spectra with reflectance spectra calculated by FDTD simulation.**

We compared Fourier power spectra and reflectance spectra calculated by FDTD simulation. The melanosome’s hexagonal structure was directional, and the direction in which the structural color was generated was the horizontal axis in Fig 5. We exported the horizontal line at the center of the vertical axis in Fig 5 and plotted the magnitude of power spectrum values with respect to wavelength, which is converted from K space using Eq. 2. Because we were only interested in the relative power for a specific value of wavelength, the absolute scale of power values was not denoted. We overlaid the predicted reflectance spectra from Fourier analysis with the reflectance spectra calculated from FDTD simulation for the comparison.


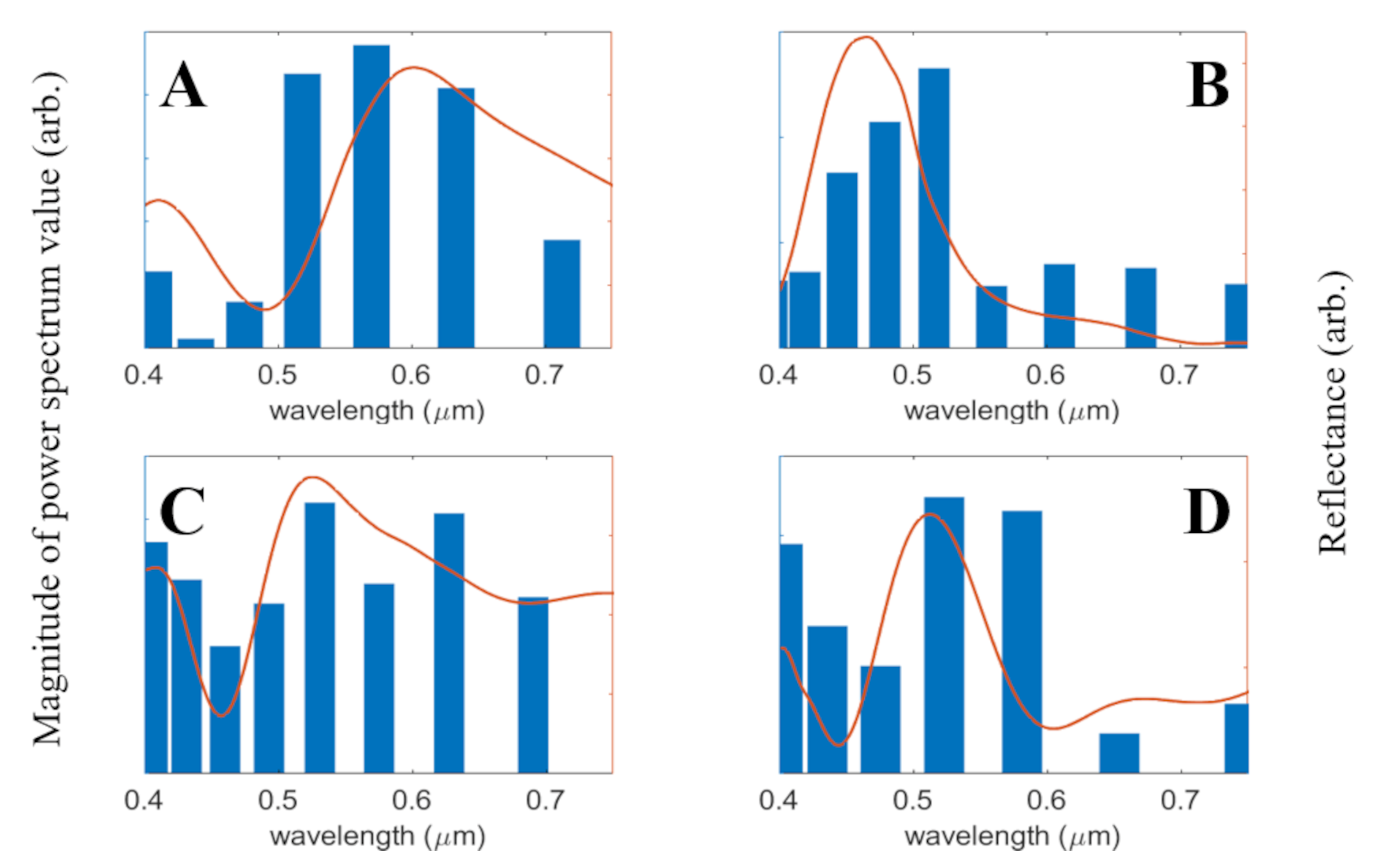


**S9 Fig. A−D Fourier power spectra (blue bars) and the reflectance spectra (orange solid lines) obtained from FDTD simulations of the sections in S3 Fig.** The expected spectra from Fourier analysis agree closely with the reflectance spectra.
